# Taxonomic studies on the genus *Isotrema* (Aristolochiaceae) from China III: *I.pseudohei*, a new species from Yunnan, Southwest China

**DOI:** 10.3897/phytokeys.186.63543

**Published:** 2021-12-06

**Authors:** Jun Wang, Guo-Dong Li, Juan-Juan Yang, Bin Shen, Chun-Xia Pu, Xin-Xin Zhu

**Affiliations:** 1 College of Life Sciences, Xinyang Normal University, Xinyang 464000, Henan, China Xinyang Normal University Xinyang China; 2 Faculty of Traditional Chinese Pharmacy, Yunnan University of Chinese Medicine, Kunming 650500, Yunnan, China Yunnan University of Chinese Medicine Kunming China; 3 Shanghai Zuibaichi Park, Shanghai 201600, China Shanghai Zuibaichi Park Shanghai China

**Keywords:** *Aristolochia*, clarification, morphology, taxonomy

## Abstract

*Isotremapseudohei*, a new species from Yunnan, Southwest China, is described and illustrated. It is morphologically similar to *I.hei* and *I.moupinense*, but differs from the former in the colour of flower and throat, the size of throat and the shape of gynostemium lobes, and from the latter in the shape of lamina and gynostemium lobes.

## Introduction

Isotrema Raf. was previously treated as a subgenus of Aristolochia L. and was recently reinstated as an independent genus base on both molecular data and morphological evidence (Zhu et al. 2019a). It can be distinguished from the genus *Aristolochia* in the strongly curved perianth with a 3-lobed limb, and especially the 3-lobed gynostemium with a pair of anthers on the outer surface of each gynostemium segment. Comprising 109 species, the genus mainly occurring in East and South Asia, with a few species disjunctly distributed in North and Central America (Zhu et al. 2019a). Currently, 71 species of *Isotrema* are recorded in China, of which 57 are endemic (Hwang et al. 2003; Lai et al. 2019; Li et al. 2019; Peng et al. 2019; Zhou et al. 2019; Zhu et al. 2019a, 2019b, 2019c, 2019d, 2019e; Cai et al. 2020a, 2020b; Luo et al. 2020; Wang et al. 2020a, 2020b; Liao et al. 2021).

During a field expedition to Yunnan, Southwest China, some unknown specimens of *Isotrema* were discovered. After careful comparison with previously known species and study of related literature (Hwang 1988; Ma 1989a, 1989b; Tao 1997; Hwang et al. 2003; Do et al. 2015; Do and Nghiem 2017; Yang et al. 2018; Zhu et al. 2019a, 2019d; Cai et al. 2020a, 2020b; Wang et al. 2020a, 2020b), we confirm it as a new species, describe and illustrate it below.

## Material and methods

Specimens of *Isotrema* from 36 herbaria (A, BM, BR, CDBI, CSFI, CSH, E, EMA, GXMI, HAST, HENU, HHBG, HIB, HITBC, IBK, IBSC, K, KUN, L, LBG, LE, NAS, NTUF, P, PE, PEM, QTPMB, SM, SNU, SYS, TAI, W, WCU, WU, WUK, YUKU; acronyms follow Thiers 2021) and our collections in the field all over Asia were examined. Meanwhile, the literature, in particular the protologues of published names, was collated and reviewed. The geographic distribution has been compiled from literatures and complemented by the analyzed specimens and color photos. Photographs of plants were taken in the wild for each species and the terminology of description was mainly based on the Flora of China (Hwang et al. 2003).

## Taxonomy

### Isotrema pseudohei

Taxon classificationPlantaeGentianalesApocynaceae

X.X.Zhu, Jun Wang bis & G.D.Li sp. nov.

D7640A65-5413-5367-9F61-378D4406FA89

urn:lsid:ipni.org:names:77234089-1

**Figure 1.**
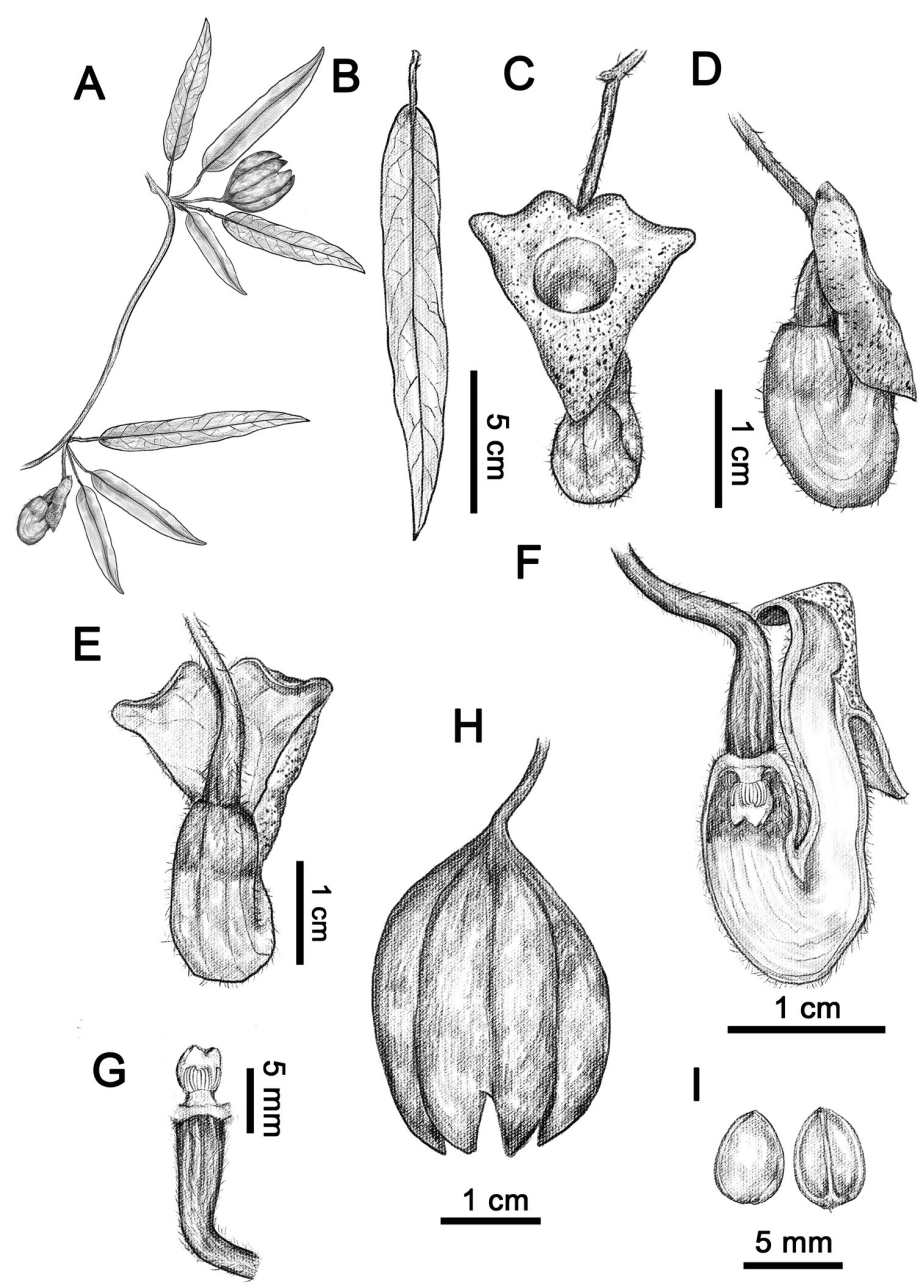
Line drawing of *Isotremapseudohei* X.X.Zhu, Jun Wang bis & G.D.Li **A** habit **B** leaf **C** flower (frontal view) **D** flower (lateral view) **E** flower (dorsal view) **F** dissected flower (showing the inside structure) **G** anthers and gynostemium **H** dehiscing capsule **I** seeds. Illustrated by Shi-Zhen Qiao.

**Figure 2.**
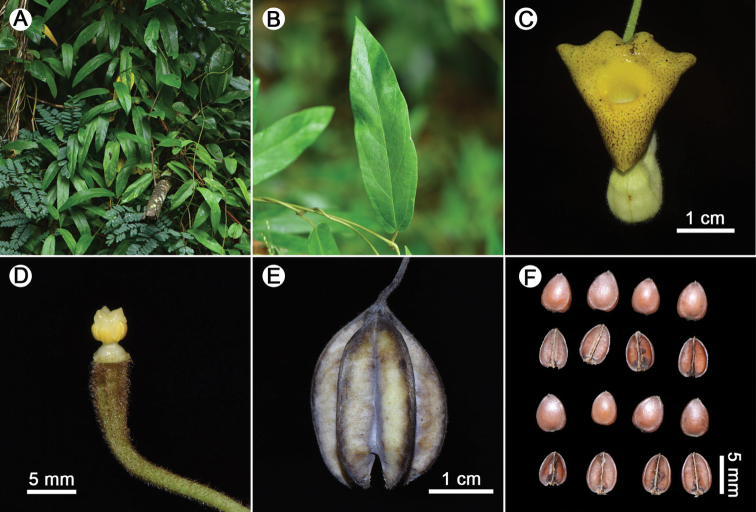
*Isotremapseudohei* X.X.Zhu, Jun Wang bis & G.D.Li from the type locality **A** habit **B** leaves **C** flower (frontal view) **D** anthers and gynostemium **E** fruit **F** seeds. Photographed by Xin-Xin Zhu.

**Figure 3.**
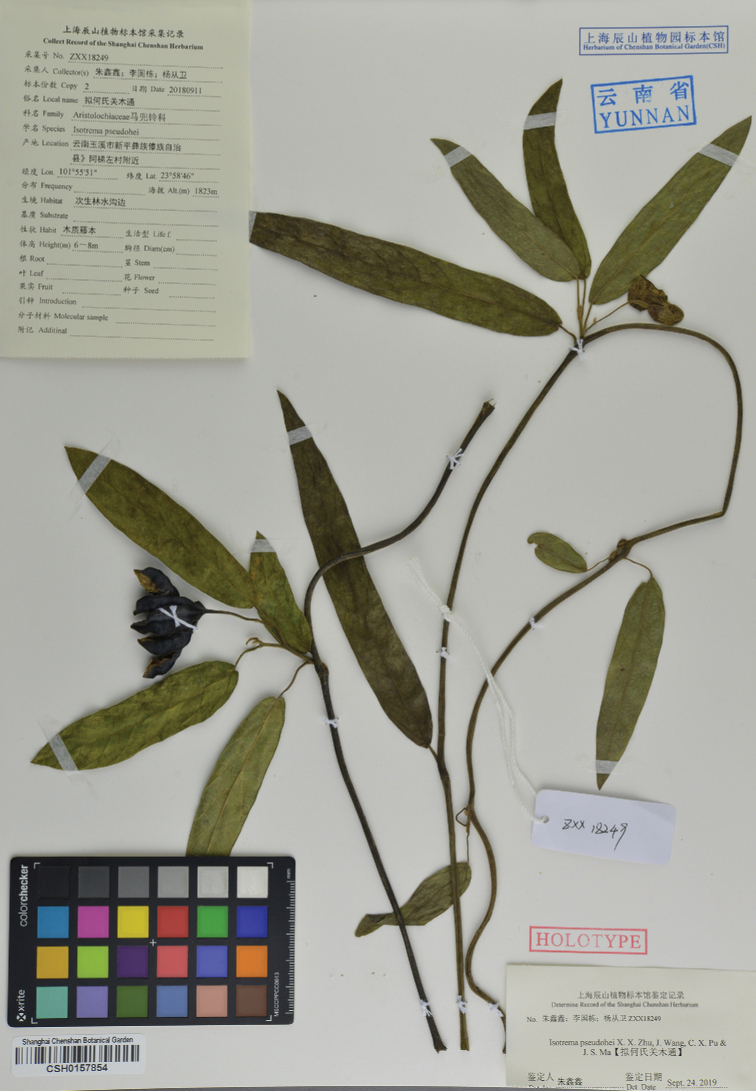
Holotype of *Isotremapseudohei* X.X.Zhu, Jun Wang bis & G.D.Li. (CSH-0157854).

**Figure 4.**
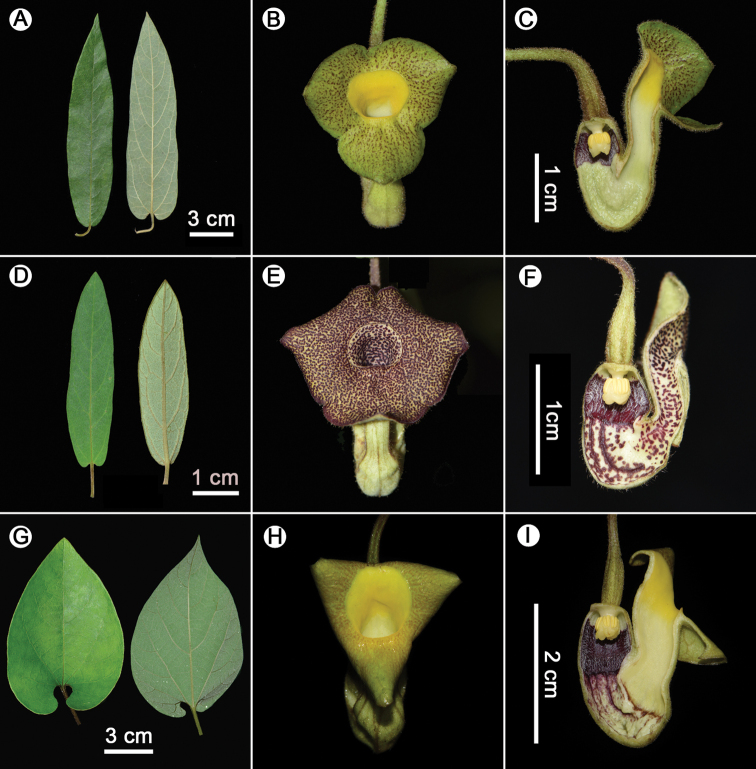
Morphological comparisons of the leaves and flowers of *Isotremapseudohei* (**A–C**), *I.hei* (**D–F**) and *I.moupinense* (**G–I**). Photographed by Xin-Xin Zhu.

Figures 1 , 2 , 3 , 4A–C

#### Type.

China. Yunnan: Yuxi City, Xinping County, near Atizuo Village, 101°55'51"E, 23°58'46"N, 1823 m a.s.l., 11 September 2018, *X. X. Zhu* et al. *ZXX18249* (holotype: CSH [CSH-0157854!]; isotypes: CSH!, KUN!).

#### Diagnosis.

Similar to *Isostremahei* Lei Cai & X.X.Zhu and *I.moupinense* (Franch.) X.X.Zhu, S. Liao & J.S.Ma, but significantly diﬀers in the following characters: laminas lanceolate to narrowly lanceolate, basal tube of calyx ca. 1.5 cm long, inside dark purple at base and yellowish white above base; upper tube of calyx ca. 2.3 cm long, inside yellowish white, getting yellow in upper portion; inner surface of limb yellow with purplish red patches; throat yellow, suborbicular, 7–9 mm in diameter; apex of gynostemium lobes acute.

#### Description.

Climbing shrubs. Stems terete, brown pubescent when young, old branchlets glabrous. Petioles 1–2 cm long, appressed villous; laminas lanceolate to narrowly lanceolate, 6.5–17.5 × 2.6–4.5 cm, base round to shallowly cordate, margin entire, apex acute, adaxially sparsely pubescent, abaxially densely villous, especially on veins, lateral veins 4–6-paired. Flower solitary, axillary or on stems; pedicels ca. 3 cm long, densely rusty villous; bractlet 1, lanceolate to elliptic, 3–4 mm long, adaxially subglabrous, abaxially densely rusty villous, inserted on middle part of pedicel. Calyx tube geniculately curved, abaxially yellowish white, densely villous; basal tube ca. 1.5 cm long, inside dark purple at base and yellowish white above base; upper tube ca. 2.3 cm long, inside yellowish white, getting yellow in upper portion; limb discoid, ca. 2.1 cm wide, shallowly 3-lobed, lobes broadly triangular, inner surface yellow with purplish red patches; throat suborbicular, yellow, 7–9 mm in diameter. Anthers 6, oblong, ca. 1.6 mm long, adnate in 3 pairs to base of gynostemium, opposite to lobes. Gynostemium 4–5 mm long, 3-lobed, apex of lobes acute. Ovary terete, ca. 8 mm long, densely brown villous. Capsule cylindric, six arrises, ca. 3 × 2.2 cm. Seeds ovate to elliptic, 3.5–5 × 3–4.5 mm, adaxially deeply concave, abaxially convex, glabrous on both sides.

#### Phenology.

Flowering and fruiting specimens of the new species were collected in September.

#### Etymology.

The specific epithet refers to the similarity between the new species and *Isotremahei* in the morphology of lamina and flower. The Chinese name is given as “拟何氏关木通”.

#### Distribution and habitat.

*Isotremapseudohei* is distributed in Xinping County of Yunnan Province, China. It grows on the roadside or in mixed forests on sunny slopes at an elevation between 1820 m and 2045 m, together with other taxa as *Castanopsis* sp. (Fagaceae), *Commelinaundulata* R. Br. (Commelinaceae), *Disporum* sp. (Colchicaceae), *Lycianthesmacrodon* (Wall.) Bitter (Solanaceae), *Rubus* sp. (Rosaceae), etc.

#### Preliminary conservation status.

*Isotremapseudohei* is currently only known from two populations in Xinping County, with fewer than five individuals at each site. Based on the present study, its Extent of Occurrence (EOO) is less than 100 km^2^ and the known Area of Occupancy (AOO) is less than 20 km^2^. Furthermore, the habitat is being destroyed by road construction and small-scale agriculture. Besides, the root of *Isotrema* itself is often harvested for medicinal purposes by the locals. Although further investigation is necessary to fully map its distribution, it is considered to be Vulnerable (VU) status, based on the criteria D2 of IUCN according to the IUCN Red List Categories and Criteria (IUCN Standards and Petitions Committee 2019).

#### Additional specimens of *Isotremapseudohei* examined (paratypes).

**China. Yunnan**: Yuxi City, Xinping County, 2045 m a.s.l., 11 September 2018, *X. X. Zhu* et al. *ZXX18250* (CSH, KUN).

#### Specimens of *Isotremahei* examined.

**China. Yunnan**: Wenshan City, 12 May 2018, *X. X. Zhu ZXX18072* (CSH), 20 April 2018, *Lei Cai* et al. *CL115* (KUN).

#### Specimens of *Isotremamoupinense* examined.

**China. Chongqing**: Nanchuan District, 27 May 1957, *T. H. Hsiung & Z. L. Chou 91052* (IBSC), 11 July 1957, *S. Y. Chen* et al. *95126* (NAS), 12 July 1957, *T. H. Hsiung & Z. L. Chou 91972* (IBSC), 25 July 1957, *T. H. Hsiung & Z. L. Chou 92285* (IBSC), 17 May 2007, *Z. B. Feng* et al. *20070508* (PE, WCSBG); **Sichuan**: Baoxing County, 4 August 1936, *K. L. Qu 3410* (IBSC), 1954, *T. P. Soong*, *38982* (IBSC, NAS), 20 May 1958, *Sichuan Agricultural College 4867* (IBSC); Dayi County, 26 May 2015, *X. X. Zhu & Z. X. Hua ZH082* (CSH), 26 May 2015, *X. X. Zhu & Z. X. Hua ZH083* (CSH); Emeishan City, 1952, *C. H. Hsiung* et al. *30475* (IBK, IBSC, NAS), 24 May 1952, *C. H. Hsiung* et al. *30750* (IBSC), 6 June 1960, *Sichuan Med. Pl. Exped. 12729* (NAS), 29 April 2015, *X. X. Zhu & Z. X. Hua ZH046* (CSH); Jinyang County, 29 April 2013, *C. Du & Y. Wang DC-325* (CSH); Luding County, 14 July 2006, *X. M. Gao G06117* (WCSBG); Yingjing County, 20 May 1940, *K. L. Chu 6901* (NAS); **Yunnan**:Gongshan County, 25 May 2011, *J. Cai* et al. *11CS2767* (KUN); Yunlong County, 24 April 2019, *X. X. Zhu* et al. *ZXX19356* (KUN).

## Discussion

**Figure 5.**
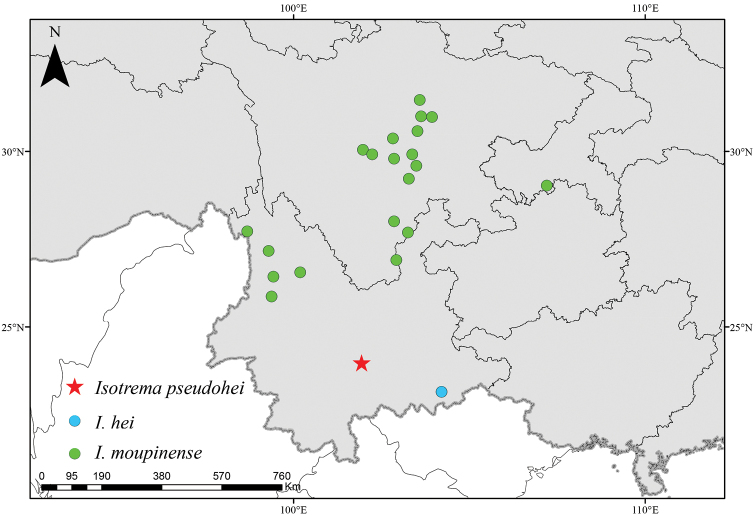
Distribution of *Isotremapseudohei*, *I.hei* and *I.moupinense* based on field observation, specimens, color photos and literatures examined.

Characterized by a horseshoe-shaped perianth and a 3-lobed gynostemium with each lobe consisting of two oblong stamens, the new species is shown to be a member of *Isotrema* (Zhu et al. 2019a). *Isotremapseudohei* is similar to *I.hei* in the shape of lamina (Fig. 4A & D), but differs in the morphology of flowers, the inner surface of limb is yellow with purplish red patches and throat suborbicular, yellow, and 7–9 mm in diameter in *I.pseudohei* (Figure 4C), whereas the inner surface of limb yellowish green and densely covered with purple papillae and throat circular, yellowish green with purple patches, and 4–5 mm in diameter in *I.hei* (Figure 4F). Though sharing similar morphology of flowers (Fig. 4B, C, H, J), *I.pseudohei* can be easily distinguished from *I.moupinense* by the shape of lamina, which is lanceolate in the new species (Figure 4A), but ovate in *I.moupinense* (Figure 4G). Moreover, *I.pseudohei* also differs in the gynostemium lobes acute at apex (Figures 2D & 4C), whereas round at apex in *I.hei* (Figure 4F) and *I.moupinense* (Figure 4I). Detailed morphological comparisons are provided in Table 1 and shown in Figure 4. Geographically, the new species is currently only known from Xinping County in central Yunnan, *I.hei* is restricted to Wenshan City in Southeast Yunnan, while *I.moupinense* is widely distributed in Southwest China (Figure 5).

**Table 1.** Morphological comparisons of *Isotremapseudohei*, *I.hei* and *I.moupinense*.

Character	*I.pseudohei*	*I.hei*	*I.moupinense*
Lamina	lanceolate to narrowly lanceolate, 6.5–17.5 × 2.6–4.5 cm	lanceolate to narrowly lanceolate or narrowly elliptic, 2.5–12.5 × 1–4.5 cm	ovate, 6–16 × 5–12 cm
Basal tube of calyx	inside dark purple at base and yellowish white above base	inside dark purple at base and yellowish white with dark purple stripes and patches above base	inside dark purple at base and yellowish with purple stripes and patches above base
Upper tube of calyx	inside yellowish white, getting yellow in upper portion	inside yellowish white with reddish purple patches	inside yellowish white, getting yellow in upper portion
Limb	ca. 2.1 cm wide; inner surface yellow with purplish red patches	ca. 2.4 cm wide; inner surface yellowish green and densely covered with purple papillae	2–2.5 cm wide; inner surface yellow to dark red, sometimes with red or yellow spots
Throat	suborbicular, yellow, 7–9 mm in diameter	circular, yellowish green with purple patches, 4–5 mm in diameter	circular, yellow, 8–11 mm in diameter
Gynostemium	apex of lobes acute	apex of lobes round	apex of lobes usually bilobate, rarely obtuse

## Supplementary Material

XML Treatment for Isotrema pseudohei
